# “Extroduction” of the Bougie for Stricture

**Published:** 1849-11

**Authors:** 


"*Extroduction*" *of the Bougie for Stricture.*—By Prof. BRAINARD. Prof. Brainard in the N. W. Med. and Surg. Journal, gives the history of a case where the patient had suffered for eleven years from stricture of the urethra following gonorrhœa; retention of urine had almost become perfect; the patient complained of great pain and a constant desire to void urine, with the ability to pass only a few drops at a time. Various careful but ineffectual attempts were made to pass the bougie, the bladder had risen several inches above the pubis, nearly to the umbilicus. The bladder was now punctured above the pubis with the long curved trochar, and the stylet withdrawn. As soon after the operation as the state of the parts would admit, long and persevering attempts to pass the bougie were made, but without success. Near the posterior extremity of the spongy portion of the urethra was a knotty projection, beyond which the instrument would not pass. The thought now occurred to Dr. B., that the prostatic and membranous portion of the urethra could be explored by means of an instrument passed into the bladder through the opening made by the puncture, and still occupied by the canula. The attempt was made by means of a small-sized bougie rendered firm by means of a stiff wire bent to form the segment of a circle. Very little difficulty was met with in the attempt to pass it. By a very little exertion the point of the instrument was brought to within two inches of the orifice of the urethra, and then seized by means of a pair of forceps, the wire was withdrawn and the open end of the bougie passed into the bladder. The instrument was allowed to remain for three days, when it was withdrawn and a larger one substituted. Larger instruments were used from time to time, until one above the medium size passed without the least difficulty. One month after the first operation the cure was nearly complete. The following practical deductions are drawn by Dr. B., from this case.

1st. The puncture of the bladder above the pubis, if care be taken to prevent infiltration of urine, is a slight operation, and should not be deferred till extreme distention takes place. In further proof of this, we would refer to a case published not long since in the Buffalo Medical Journal, by our friend, Dr. J. P. White, of that city. In that case a puncture of the kind served as a substitute for a urethra for a long time with but trifling inconvenience.

2d. This puncture may be made useful in catheterism. For this operation of passing a catheter forwards, which, so far as we are aware, has not been done before, a friend has suggested the name of *Extroduction.* It may be performed with a properly curved instrument very readily, and if difficulties should occur, they may be obviated by passing the finger into the rectum. If the cases in which it is likely to be useful are rare, they are extremely urgent, and when they occur, this operation may prove a valuable means of relief.—*N. Y. Journal of Medicine.*

